# Novel HPLC–laser-stimulated fluorescence profiling of seminal plasma amino acids linked to ejaculate quality

**DOI:** 10.1007/s10815-026-03944-9

**Published:** 2026-07-02

**Authors:** Megha Naik, Huidrom Yaiphaba Meitei, Shubhashree Uppangala, Dhakshanya Predheepan, Satish Kumar Adiga, Ajeetkumar Patil

**Affiliations:** 1 Manipal Institute of Applied Physics, Manipal Academy of Higher Education, Manipal, 576104 India; 2 SAR Healthline, Private Ltd., Chennai, India; 3 Division of Reproductive Genetics, Department of Reproductive Science, Kasturba Medical College, Manipal Academy of Higher Education, Manipal, 576104 India; 4 Centre of Excellence in Clinical Embryology, Department of Reproductive Science, Kasturba Medical College, Manipal Academy of Higher Education, Manipal, India; 5 Division of Clinical Embryology, Department of Reproductive Science, Kasturba Medical College, Manipal Academy of Higher Education, Manipal, India

**Keywords:** Amino acid, Biomarker, Male infertility, Seminal plasma, Spermatozoa

## Abstract

**Purpose:**

Amino acids are vital biomolecules that can provide important insights into sperm functional competence. However, the level of amino acids (AAs) in seminal plasma and their implications on sperm characteristics remain less understood.

**Methods:**

For the first time, high-performance liquid chromatography (HPLC) in combination with a laser-stimulated fluorescence (LSF) system was used to profile AAs from the seminal plasma of 30 normozoospermic and 51 nonnormozoospermic ejaculates. Sperm characteristics, along with DNA integrity, were assessed and correlated with individual AAs.

**Results:**

The HPLC-LSF system was able to detect a total of 18 AAs in the seminal plasma. A significant variation was observed between the two groups for all AAs (*p* < 0.05), except for aspartate. Notably, the relative intensities of AAs were significantly lower in the non-normozoospermic group. Principal component analysis (PCA) and partial least squares discriminant analysis (PLS-DA) further highlighted distinct clusters based on the AA profiles of the two groups. The pathway analysis indicated differences in the metabolic pathways between the normozoospermic and nonnormozoospermic groups.

**Conclusion:**

The HPLC-LSF platform could serve as a potential tool in identifying seminal plasma AA levels. The low relative abundance of AA in the non-normozoospermic men could be attributed to aberrant metabolic pathways, underscoring their significance in male infertility.

**Supplementary Information:**

The online version contains supplementary material available at https://doi.org/10.1007/s10815-026-03944-9.

## Introduction

Human seminal plasma comprises several metabolites such as glucose, fructose, cholesterol, proteins, amino acids (AAs), antioxidant enzymes, and minerals crucial for sperm metabolism and function [1]. Among these, AAs are crucial metabolites for male reproductive health, and their presence in seminal fluid is essential for optimal sperm function [2]. In addition to being the building blocks of proteins, AAs serve several important nonprotein functions, such as scavengers for free radicals, protecting cells from the damaging effects of osmolality, while also functioning as buffers to protect sperm cells and provide oxidizable substrates to support sperm metabolism [3]. Studies have revealed that the metabolism and motility of sperm are significantly influenced by certain AAs found in seminal plasma [4]. Furthermore, it was observed that adding AA supplements to the diet enhances the quality of the sperm, which in turn enhances the ability to fertilize [5]. Importantly, recent research has highlighted the potential of metabolic profiling of seminal plasma as an approach to elucidate the molecular mechanism regulating sperm function and overall reproductive competence [6–9]. Thus, AA analysis in seminal plasma is crucial to understanding the pathophysiological process behind male infertility.

To achieve reliable metabolite profiling in complex biological fluids such as seminal plasma, high-sensitivity analytical techniques are essential. However, accurately detecting low-abundance metabolites can be challenging due to matrix complexity and the limitations of conventional analytical methods [10]. In this context, high-performance liquid chromatography combined with the laser-stimulated fluorescence (HPLC-LSF) technique was evaluated as a sensitive analytical tool for profiling AAs in human seminal plasma. It requires only trace amounts of samples and allows for better selective separation of AAs. HPLC-LSF can simultaneously detect 20 AAs at femtomole sensitivity and has proven to be effective and dependable, providing a comprehensive examination of the AA levels in biological fluids [11]. Additionally, the AAs in seminal plasma were analyzed to assess the differences in their relative abundances (signal intensities) between normozoospermic and non-normozoospermic samples, providing insights into possible mechanisms associated with male infertility.

## Materials and methods

### Study subjects and design

Semen samples obtained from men referred to the Andrology Laboratory, Kasturba Medical College, Manipal, for infertility screening between 2020 and 2023 were included in this study. Samples were categorized as normozoospermic (*n* = 30) and non-normozoospermic (*n* = 51) based on primary semen analysis. The non-normozoospermic group includes oligozoospermic (*n *= 33), oligoasthenozoospermic (*n *= 11), and oligoasthenoteratozoospermic (*n *= 07) samples. Fertility status of the female partner and primary, secondary, or other level of investigation of men was not performed.

The study was conducted in accordance with the Declaration of Helsinki for conducting research involving humans, and it was approved by the Institutional Ethics Committees (IEC 548/2020). All the participants included in this study have given written informed consent. Patients were instructed to maintain 2–7 days of ejaculatory abstinence. Semen analysis was performed as per the WHO criteria (WHO, 2010) [12]. Post analysis, seminal plasma was separated by centrifugation (1000 g for 15 min) and stored at − 80 °C until AA estimation using the HPLC-LSF system.

### Assessment of sperm characteristics

Post liquefaction, the sperm concentration was determined using a Makler counting chamber (Sefi Medical Instruments Ltd., Israel) with adequate quality control measures. Sperm motility was assessed on a microscopic slide, and a minimum of 200 cells were scored with 40 × magnification. The vitality was measured using the eosin-nigrosine method, whereas morphology was assessed in the Shorr-stained slides. A checklist is provided in the supplementary information where deviations from the recommended semen analysis methods were stated (Supplementary information).

### Sperm chromatin dispersion (SCD) test

The SCD test was conducted following the protocol outlined previously [13]. Approximately 0.5–1 million sperm cells were mixed in 1% of low-melting-point agarose warmed at 37 °C. The sperm suspension was overlaid on a precoated slide with 0.65% normal melting point agarose, followed by solidification. The slides were immersed in 0.08 N hydrochloric acid for 7 min at room temperature for denaturation. The denatured cells were subjected to lysis solution 1 (0.4 M tris HCl, 50 mM EDTA, 1% SDS, 20 mM DTT), lysis solution 2 (0.4 M tris HCl, 2 M NaCl), neutralization solution (0.4 M tris HCl), and serial dehydration with different concentrations of ethanol. The assessment was performed using a fluorescent microscope (Imager-A1, Zeiss, Gottingen, Germany) after staining the cells with ethidium bromide (7 µg/mL). A total of 500 cells were assessed, and the percentage of DNA fragmentation of spermatozoa was determined and classified by quantifying sperm cells exhibiting halos [14, 15]. Specifically, the percentage of DNA-fragmented spermatozoa was calculated by counting spermatozoa with a small + no halo, and the percentage of spermatozoa with severely damaged DNA was calculated by counting spermatozoa with no halo, whereas those with a large halo were considered to have intact DNA and thus categorized as normal.

### Amino acid profiling using HPLC-LSF system

#### Sample preparation

A chromatogram of standard AAs was recorded before analyzing the seminal plasma to aid in their identification by providing reference retention times and spectral data. A known concentration of the standard AA mixture was prepared using a sodium bicarbonate buffer with a pH of 9.2. To this solution, 5 µL of Dns-Cl (37 mM), prepared in ACN, was added. The mixture was then allowed to sit at room temperature for 1 h, after which the excess Dns-Cl was quenched with an NH_4_OH solution. The detailed derivatization procedure is discussed earlier [11]. To record the AA profiles of seminal plasma samples, each sample was diluted at a ratio of 1:300 using bicarbonate buffer. The diluted sample was then derivatized with Dns-Cl and then injected into the HPLC system for analysis.

#### HPLC-LSF conditions

The HPLC-LSF system comprises two parts: one being the AA separation part and the other being the detection part. The dansylated AAs were separated using an HPLC system (Agilent 1200 series) equipped with a Rheodyne 7725i manual injector with a 20 µL loop, followed by a reverse-phase C18 column (5 μm, 250 mm × 4.6 mm i.d., Agilent, USA, No. 770995–902). For the separation of AAs, mobile phase A comprises a sodium acetate buffer (83 mM, pH 5.94), while mobile phase B consists of methanol. The AAs were eluted under a gradient, beginning with 60% solvent A, decreasing to 55% over 26 min, 40% over 35 min, and finally reaching 0% in 60 min. After separation, the Dns-AAs eluting through a UV-grade quartz capillary (75-μm inner diameter) were excited using a 325-nm He-Cd laser. The emitted fluorescence signal from Dns-AAs was collected at a right angle and focused on the monochromator slit set at 530 nm. The signal was collected by a photomultiplier tube connected to the monochromator, operated at − 850 V. The signals were further amplified using a preamplifier and a lock-in amplifier (LA). The chromatogram was generated using a computer connected to an LA. The HPLC-LSF approach used for analyzing AAs has been described and validated for its sensitivity, accuracy, and precision [11]. The precision of the method was evaluated by repeatedly analyzing AA standards, and the relative standard deviation (RSD) obtained was less than 5%. This indicates good intraday and interday reproducibility. To assess the method’s accuracy, calibration curves were generated using a standard AA mixture, which showed good linearity over the tested range (*R*^2^ > 0.98). The method demonstrated high sensitivity, with limit of detection (LOD) values ranging from 4.3 to 85.3 fmol. This validated method was subsequently applied to analyze AAs in seminal plasma samples.

### Statistical analyses

Post AA profiling, the chromatograms were subjected to preprocessing, such as baseline correction, smoothing, *x*-axis shifting, and interpolation, to minimize run delay, noise removal, and unwanted background using GRAMS/32 (Galactic Inc., USA) as described earlier [16–19].

The statistical analysis was conducted using Jamovi 2.5 and MetaboAnalyst 6.0 (https://www.metaboanalyst.ca/) [20, 21]. The comparison of AAs between the group of samples was performed using either an independent *t*-test or the Mann–Whitney test according to the data distribution. This univariate analysis was performed to detect AAs with significant differences between normozoospermic and nonnormozoospermic groups. Additionally, correlation analysis was carried out to examine the relationship between individual AAs and sperm parameters. The Spearman’s rank correlation coefficient was selected as the statistical measure for this analysis. The results provided the correlation coefficients (*r*) and significance levels, enabling the identification of AAs that are either positively or negatively associated with sperm parameters. Multivariate analysis, such as principal component analysis (PCA) and partial least squares discriminant analysis (PLS-DA), was used to gain insights into the overall metabolic changes. To evaluate the impact of individual features on group differentiation, the variable importance in projection (VIP) scores from the PLS-DA model were calculated. A higher VIP score indicates a greater influence on the model. Generally, factors with VIP scores above 1 are considered significant contributors. Receiver operating characteristic (ROC) curve analysis was used to evaluate the diagnostic accuracy of the tests. Additionally, pathway analysis was utilized as a tool to explore potential biological mechanisms that may be associated with altered AA metabolism in the context of male infertility.

## Results

### Semen characteristics

Semen characteristics of the study subjects are available in the supplementary data (Supplementary Table S1).

### Detection of AA levels in seminal plasma

**Fig. 1:**
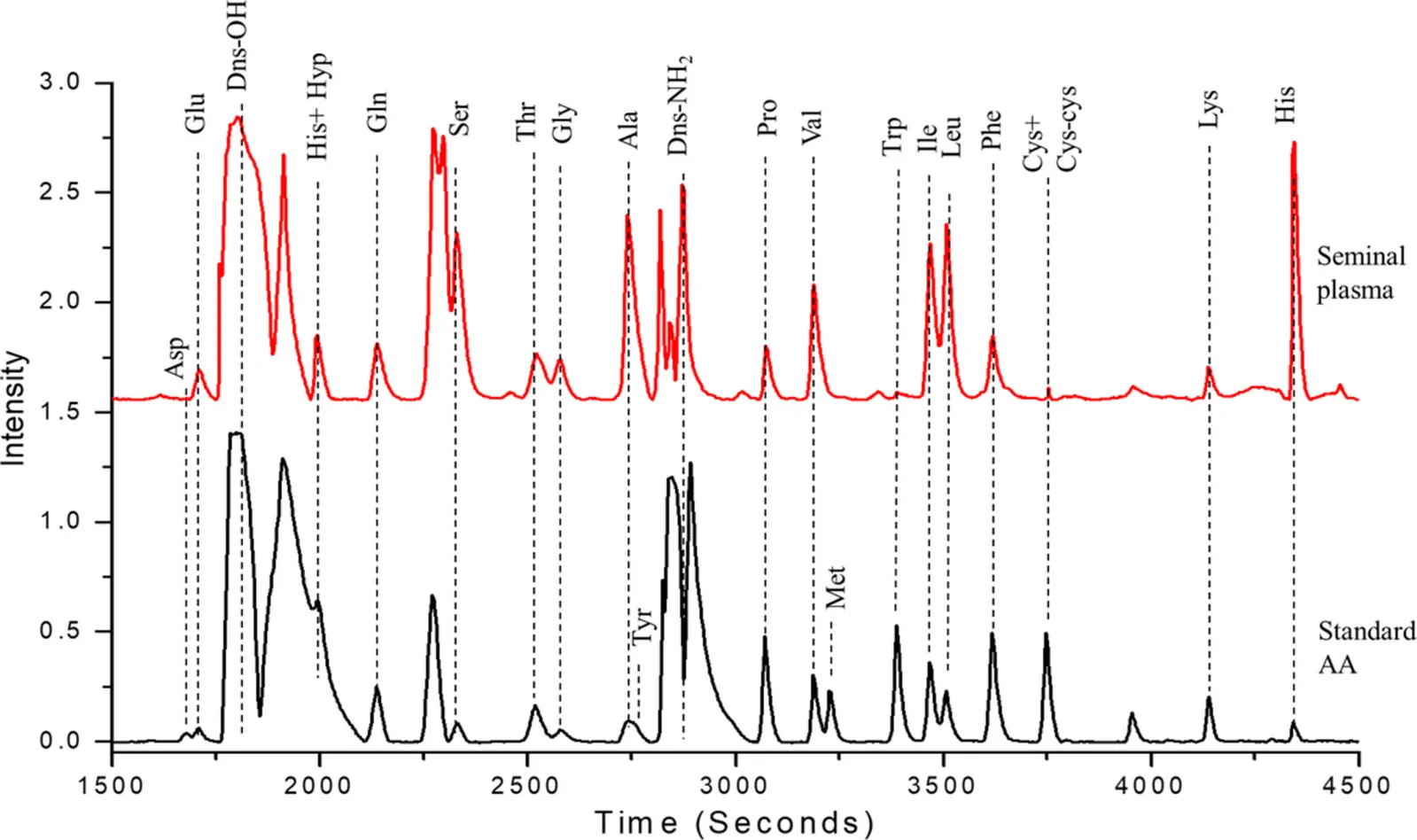
Comparison of the chromatogram of a standard AA mixture and a typical seminal plasma chromatogram

A total of 18 AAs were detected in the seminal plasma. A typical chromatogram of a seminal plasma sample, alongside the chromatogram of standard AAs, is shown in Fig. 1. This comparative representation facilitates the accurate identification of the AAs present in the seminal plasma.

### Comparison of AA intensity between normozoospermic and non-normozoospermic groups

The signal intensities of all detected AAs in the seminal plasma of normozoospermic and non-normozoospermic samples were compared to identify any differences between the two groups. Due to the limited number of samples in each category, particularly in the oligoasthenozoospermic and oligoasthenoteratozoospermic groups, the subgroups were combined into a single non-normozoospermic group to ensure sufficient statistical power. The Mann–Whitney *U* test, which is a nonparametric alternative to the independent *t*-test, was used for this comparison because the data were not normally distributed. Table 1 displays the differences in AA intensities between normozoospermic and non-normozoospermic samples. All AAs exhibited significant differences between the two groups, except for aspartate. The peak intensities of the other 17 AAs were significantly lower in the non-normozoospermic samples compared to the normozoospermic samples (*p* < 0.001).

**Table 1:** Intensity variation of AA peaks between normozoospermic and non-normozoospermic samples

Amino acids	Normozoospermic (*n* = 30)		Nonnormozoospermic (*n* = 51)		*p* value	Effect size
	Median	IQR	Median	IQR		
Aspartate	0.01	0	0.01	0.01	0.071	0.0408
Glutamate	0.2	0.0725	0.12	0.08	< 0.001	0.3923
Hydroxyproline	0.32	0.0875	0.2	0.11	< 0.001	0.4605
Glutamine	0.245	0.075	0.15	0.12	< 0.001	0.436
Serine	0.74	0.103	0.5	0.21	< 0.001	0.6376
Threonine	0.25	0.08	0.16	0.09	< 0.001	0.5033
Glycine	0.18	0.035	0.11	0.06	< 0.001	0.5607
Alanine	0.775	0.14	0.54	0.19	< 0.001	0.6291
Proline	0.295	0.095	0.18	0.07	< 0.001	0.3508
Valine	0.6	0.14	0.36	0.2	< 0.001	0.6018
Tryptophan	0.035	0.01	0.02	0.01	< 0.001	0.2416
Isoleucine	0.705	0.16	0.5	0.15	< 0.001	0.5938
Leucine	0.845	0.165	0.57	0.26	< 0.001	0.5888
Phenylalanine	0.33	0.0675	0.2	0.14	< 0.001	0.5278
Cysteine + cystine	0.02	0.01	0.01	0	0.005	0.098
Lysine	0.19	0.0475	0.09	0.08	< 0.001	0.4088
Histidine	0.945	0.185	0.54	0.25	< 0.001	0.5484

*IQR* interquartile range

To account for possible phenotype-specific variations, the exploratory analyses were conducted separately for each non-normozoospermic subgroup. The related data are presented in Supplementary Table S2. The median intensities of several amino acids were generally lower in the infertile groups compared to the normozoospermic group. The observed decrease was significant across all groups (O, OA, and OAT) when compared to the normozoospermic group for the following amino acids: glutamate, hydroxyproline, glutamine, serine, threonine, glycine, alanine, proline, valine, isoleucine, leucine, phenylalanine, lysine, and histidine. Certain amino acids exhibited subgroup-specific variations. For instance, aspartate showed significant differences between the normozoospermic group and the oligoasthenozoospermic and oligoasthenoteratozoospermic groups. Furthermore, tryptophan displayed significant differences between the normozoospermic, oligozoospermic, and oligoasthenoteratozoospermic groups. In contrast, cysteine + cystine showed significant variation only between normozoospermic and oligozoospermic samples. These results suggest that while some metabolic changes are consistent across all fertility phenotypes, specific alterations in amino acid levels may vary depending on the severity and combination of sperm defects.

**Fig. 2:**
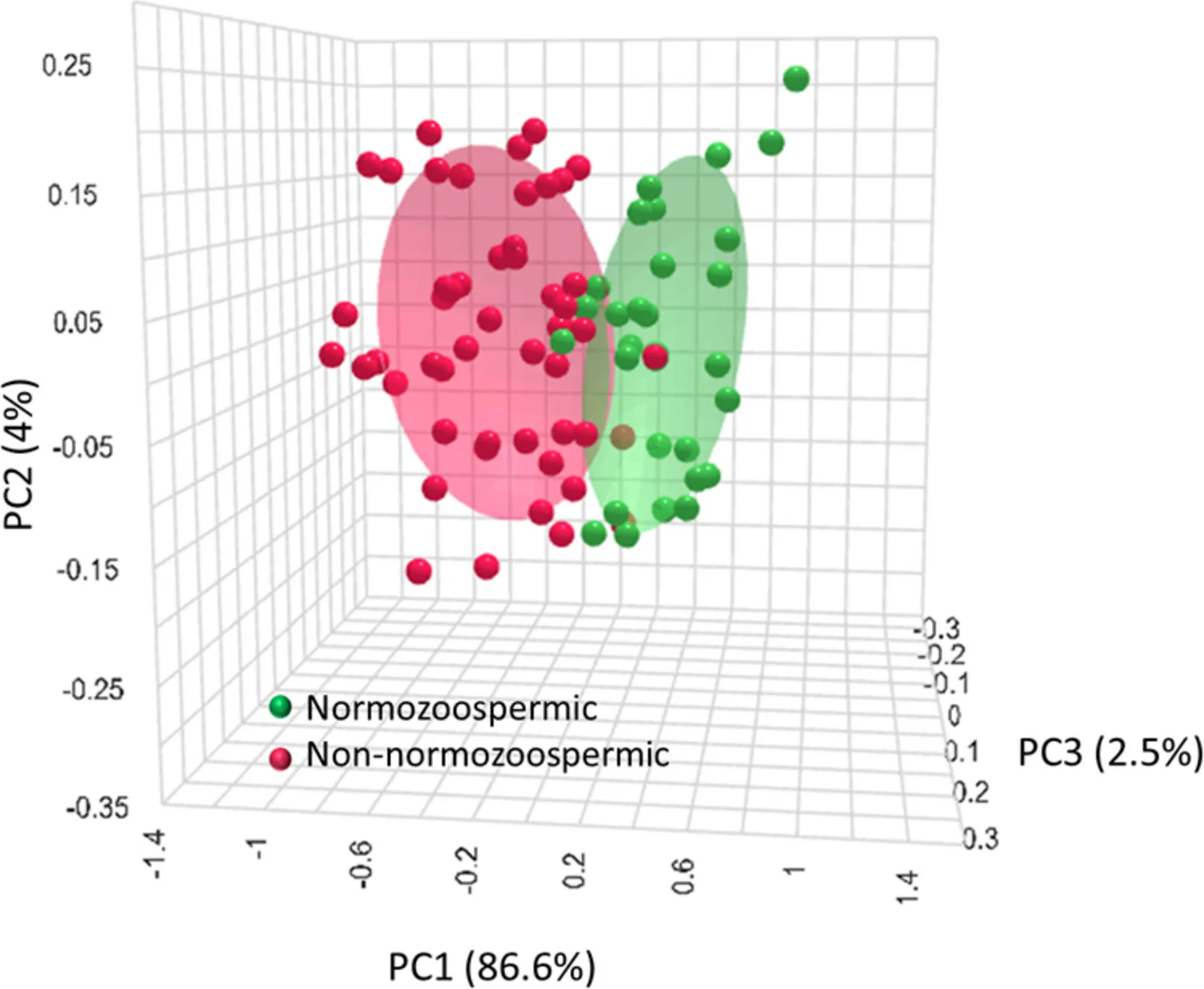
PCA 3D scatter plot of seminal plasma AAs. Green indicates normozoospermic samples and pink indicates non-normozoospermic samples. The shaded ellipsoids represent the 95% confidence interval for each group based on PCA scores derived from AA peak intensities

**Fig. 3:**
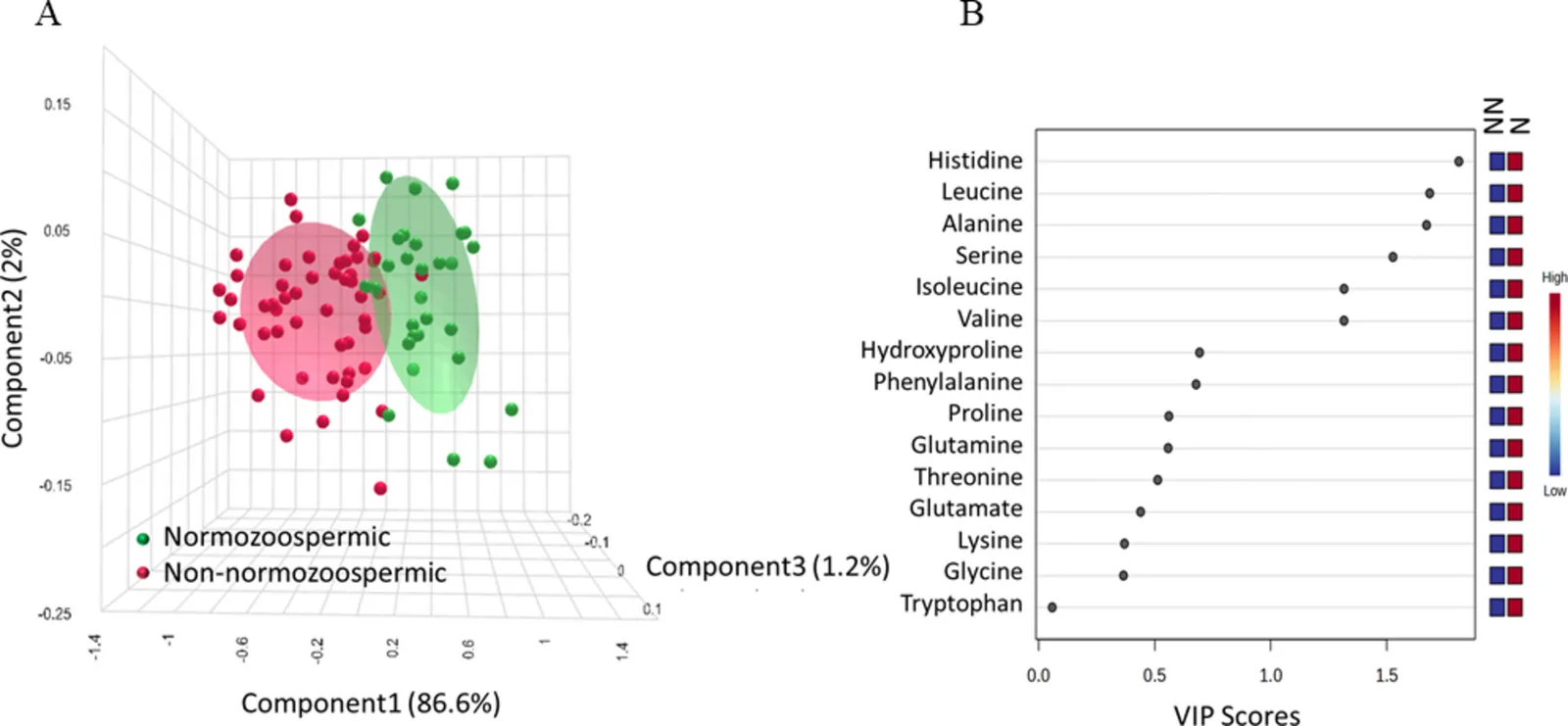
**A** PLS-DA score plot exhibiting separation between N and NN samples. **B** VIP score plot obtained from PLS-DA highlighting AA contribution to group discrimination (VIP > 1) (normozoospermic (N) group; nonnormozoospermic (NN) group)

A multivariate analysis was performed to evaluate potential correlations among metabolites and their relationship with male infertility conditions. The metabolic changes among the sample cohorts were visualized using PCA and PLS-DA. Figure 2 shows a 3D PCA score plot, where principal components 1, 2, and 3 accounted for 86.6%, 4%, and 2.5% of the variance, respectively. A distinct separation between the groups was observed. To further investigate the differences between the two cohorts and identify the AAs responsible for the observed separation trend, PLS-DA was employed. Figure 3a, b represents the 3D PLS-DA model and the VIP score plot for the seminal plasma AAs of normozoospermic and non-normozoospermic groups, respectively. The PLS-DA model showed improved separation between the two groups, with several AAs exhibiting VIP scores greater than 1. Notably, branched-chain amino acids (BCAAs), along with alanine, serine, and histidine, all demonstrated VIP scores greater than 1, highlighting their important role in differentiating these two groups.

Individual analyses, such as PCA and PLS-DA, were also conducted to evaluate metabolic differences among subgroups of male infertility. The outcomes specific to each group are detailed in the Supplementary Data (Fig. S1, S2, S3, S4). The pairwise comparisons revealed that some oligozoospermic samples overlapped with normozoospermic samples; however, oligoasthenozoospermic and oligoasthenoteratozoospermic samples were distinctly separated from normozoospermic samples. It is noteworthy that the subgroup analyses yielded AA VIP score patterns for the PLS-DA models that closely matched those observed in the comparison between normozoospermic and non-normozoospermic samples. This indicates that the discriminating AAs remained consistent across both subgroup analyses and the combined-group analysis.

### Correlation analysis

**Fig. 4:**
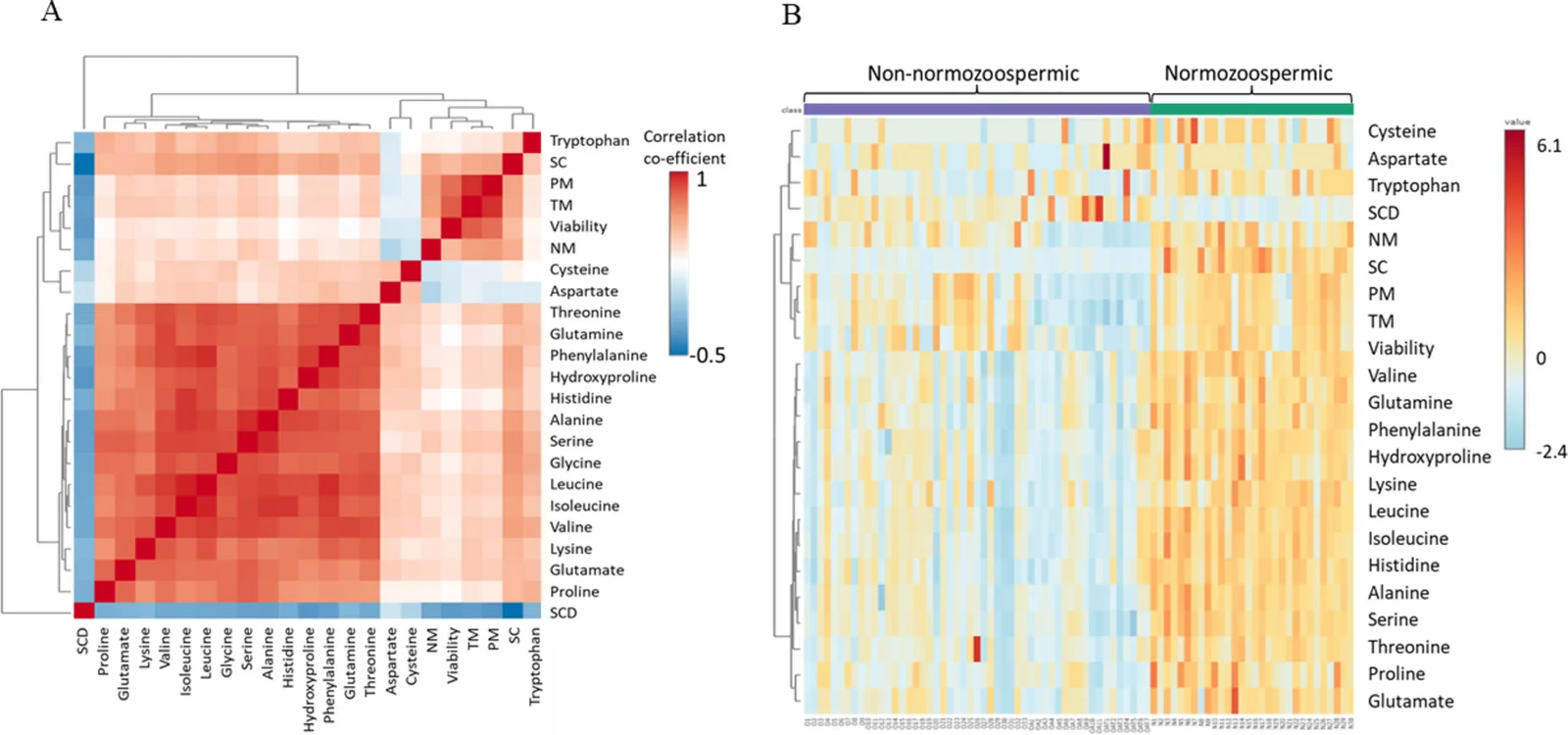
**A** Heatmap correlation between sperm parameters and AAs in seminal plasma. **B** Heatmap visualization of variations in seminal plasma AA intensities across individual samples

The relative intensities of AAs were correlated with the sperm characteristics such as concentration, motility, morphology, viability, and DNA integrity. Spearman correlation analysis was conducted to evaluate the clinical significance of these AAs. Figure 4a presents a correlation heatmap displaying the association between AAs and sperm characteristics. A moderate positive correlation was observed between sperm concentration and several AAs: proline (*r* = 0.51), glutamate (*r* = 0.51), lysine (*r* = 0.53), valine (*r* = 0.62), isoleucine (*r* = 0.60), leucine (*r* = 0.62), glycine (*r* = 0.64), serine (*r* = 0.66), alanine (*r* = 0.62), histidine (*r* = 0.53), hydroxyproline (*r* = 0.58), phenylalanine (*r* = 0.59), glutamine (*r* = 0.50), and threonine (*r* = 0.56) with all correlations exhibiting a *p* value < 0.001. These correlations indicate the important role of these AAs in sperm development and metabolism.

Conversely, a weak negative correlation was observed between valine (*r* =  − 0.30), isoleucine (*r* =  − 0.30), leucine (*r* =  − 0.31), glycine (*r* =  − 0.32), serine (*r* =  − 0.34), alanine (*r* =  − 0.35), and phenylalanine (*r* =  − 0.36) with SCD test results (*p* < 0.01). Additionally, certain AAs such as glutamate (*r* = 0.45), lysine (*r* = 0.45), valine (*r* = 0.43), leucine (*r* = 0.43), serine (*r* = 0.46), alanine (*r* = 0.42), and threonine (*r* = 0.45) demonstrated a moderate positive correlation with sperm motility. Serine (*r* = 0.45) has shown a moderate positive correlation with normal sperm morphology, and all these AAs exhibited a significance level of *p* < 0.01.

The heatmap shown in Fig. 4b illustrates the variations in sperm parameters and AA content between normozoospermic and non-normozoospermic samples. Each row represents specific AAs and sperm parameters, while each column corresponds to individual samples. The intensity of red and blue in the heatmap indicates these variables’ relative concentrations or correlations; red signifies higher levels of AAs (positive values), and blue indicates lower levels of AAs (negative values). The figure reveals that non-normozoospermic samples exhibited lower levels of AAs, suggesting significant metabolic alterations.

### Receiver operating characteristic (ROC) curve analysis

A ROC curve was plotted to evaluate the diagnostic accuracy of significant AAs in differentiating between normozoospermic and non-normozoospermic conditions. This curve demonstrates the model’s ability to distinguish between the two groups by comparing the true and false positive rates. The ROC curve was generated using Monte Carlo cross-validation (MCCV) with balanced subsampling. In MCCV, two-thirds of the samples were utilized to assess feature importance, and a classification model was constructed based on these important features. The model was then validated using the remaining one-third of the samples. The ROC curve was analyzed using the class probabilities from the PLS-DA model, which was constructed with a single latent component that demonstrated the best predictive capability (*Q*^2^). For both feature ranking and classification, the PLS-DA model was employed.

**Fig. 5:**
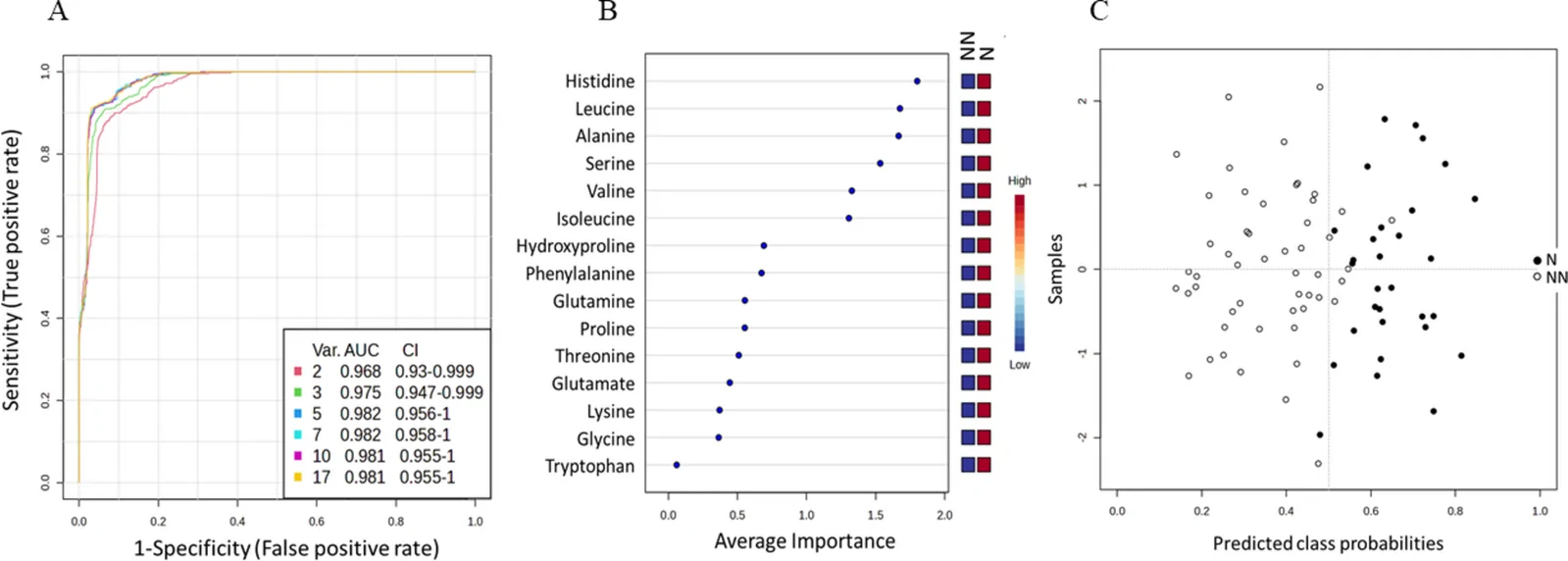
**A** Receiver operating characteristic (ROC) curve illustrating the classification performance of the multivariate model. **B** Ranking of AAs based on mean importance values contributing to the predictive model. **C** Predicted class probabilities for normozoospermic and non-normozoospermic samples derived from the classification model

Figure 5a, b shows the ROC curve and the metabolite importance plot, which classify normozoospermic and non-normozoospermic samples based on the relative abundance of AAs in seminal plasma. A combination of five features achieved an area under the curve (AUC) of 0.96, indicating excellent diagnostic accuracy. In Fig. 5b, 15 AAs are ranked according to their contribution to the classification model, with histidine, leucine, alanine, serine, and valine identified as the most important metabolites.

Figure 5c displays the predicted class probabilities for normozoospermic and nonnormozoospermic samples. The *x*-axis shows the likelihood of a sample being classified as either normozoospermic or non-normozoospermic, while the *y*-axis represents the individual samples. This visualization helps to clarify how effectively the model categorizes the samples into their respective groups. The sensitivity and specificity were calculated from the confusion matrix, yielding 88.2% and 96.6%, respectively. Subgroup analyses were performed for each individual non-normozoospermic phenotype. All subgroup models displayed similar discriminatory performance, with AUC values exceeding 0.96 (Fig. S5, S6, S7).

### Pathway analysis

**Fig. 6:**
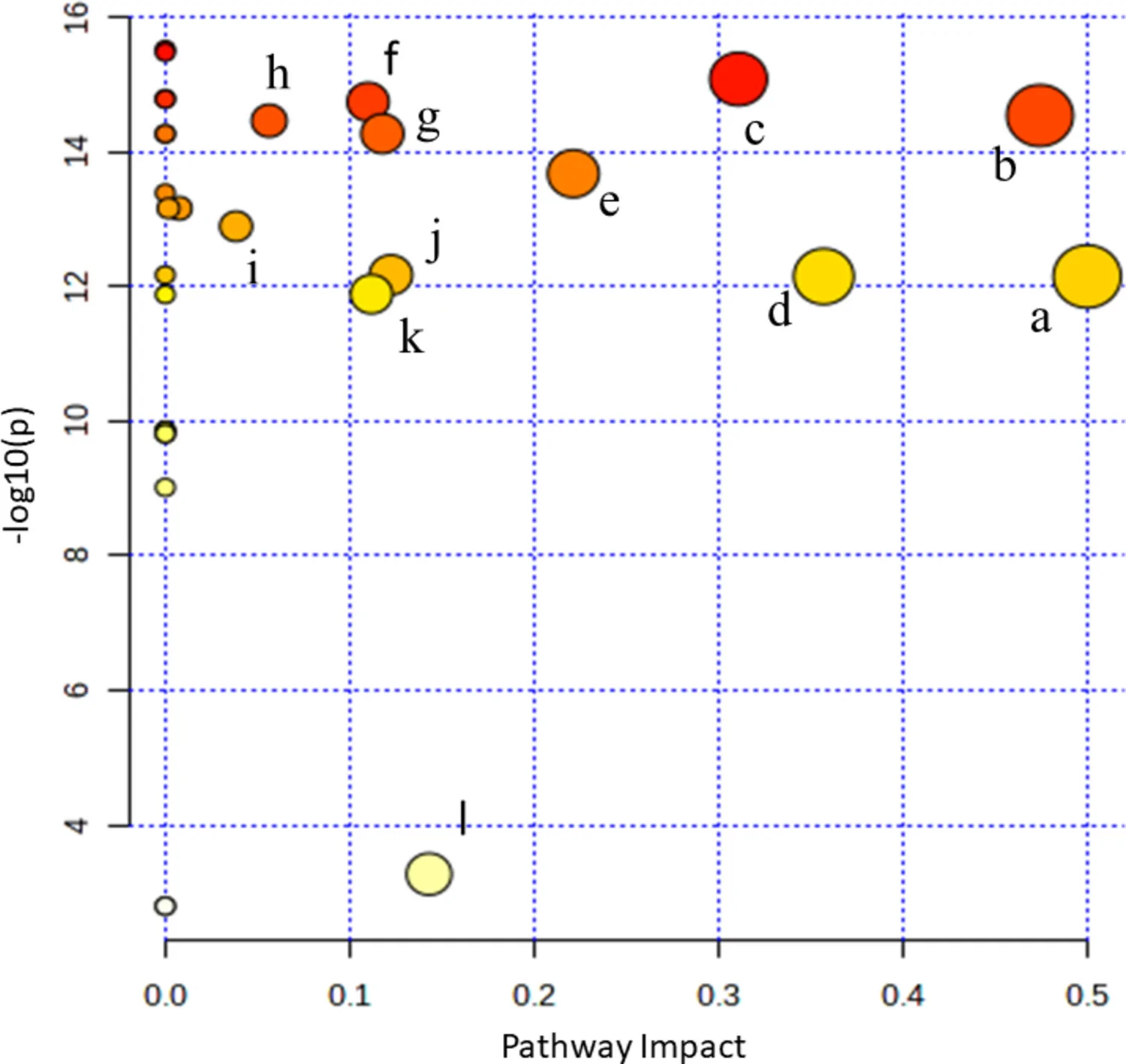
Overview of pathway analysis based on enrichment analysis procedure

The results of the pathway analysis summarize the significantly altered metabolic pathways based on the AA levels found in the seminal plasma of both normozoospermic and non-normozoospermic samples. Among these results, the metabolic pathways that were most affected are highlighted in red, while those that were less affected are highlighted in shades of orange to yellow. Figure 6 presents the Kyoto Encyclopedia of Genes and Genomes (KEGG) pathway enrichment bubble graphic, illustrating the significant metabolic pathways that have been enriched. Based on the findings, ten pathways were identified as statistically significant and biologically impactful. These include arginine biosynthesis (impact 0.12), phenylalanine, tyrosine, and tryptophan biosynthesis (impact 0.5), histidine metabolism (impact 0.22), cysteine and methionine metabolism (impact 0.11), alanine, aspartate, and glutamate (impact 0.31), phenylalanine metabolism (impact 0.36), glyoxylate and dicarboxylate metabolism (impact 0.11), glycine, serine, and threonine metabolism (impact 0.47), glutathione metabolism (impact 0.11), and tryptophan metabolism (impact 0.14).

**Fig. 7:**
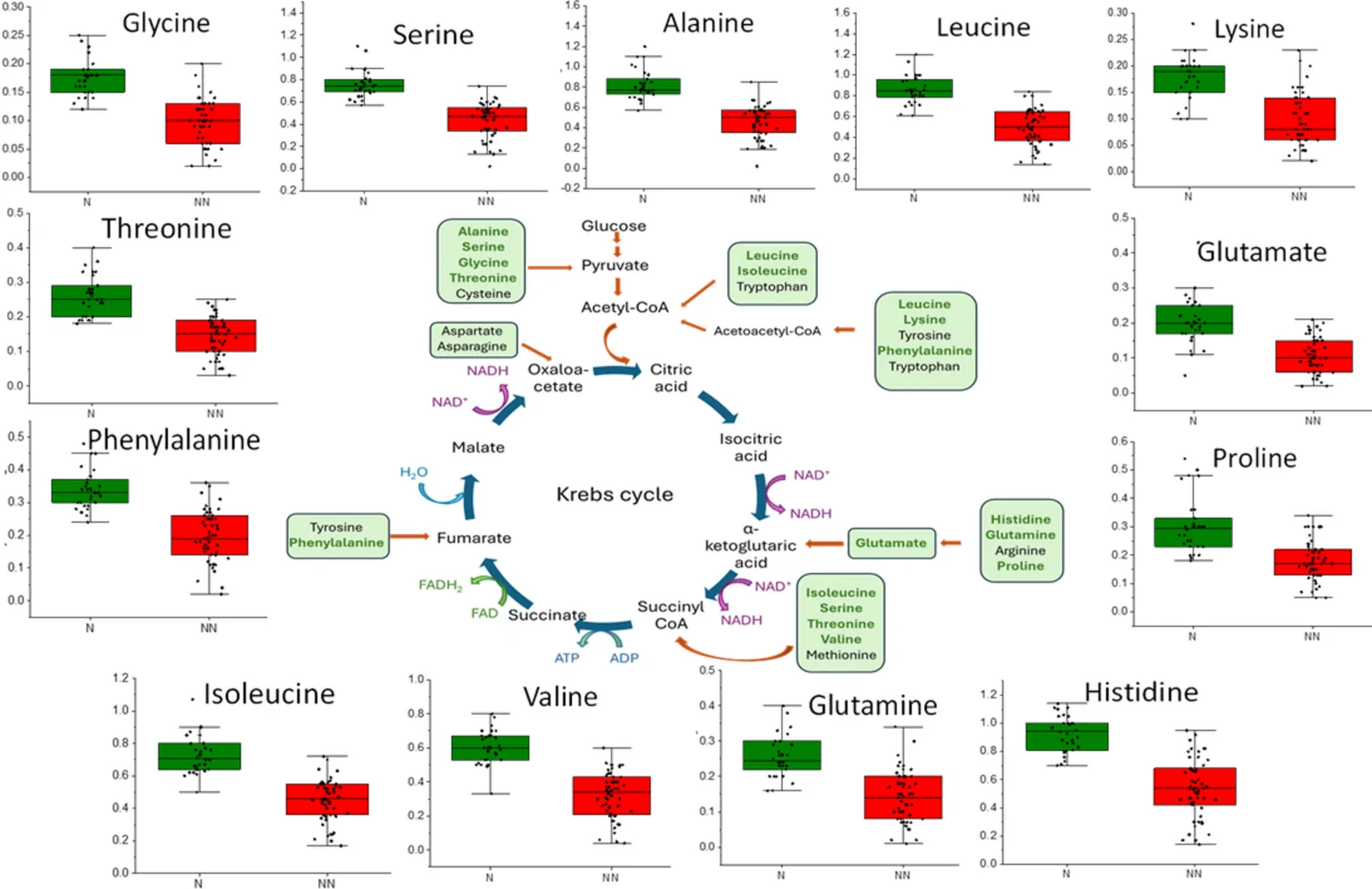
Metabolic network of the AAs with significant changes. AAs that have been upregulated are shown in green (fold change 1.6). Box plots depict relative intensities of AAs in normozoospermic (N) and non-normozoospermic (NN) groups with statistical significance indicated (*p* < 0.001)

A metabolic network was constructed to visualize the altered AA profiles, where the upregulated AAs are highlighted in green in the Krebs cycle (Fig. 7). Thirteen AAs were shown to be upregulated based on the fold change (FC) analysis. This network would help delineate some of the important metabolic pathways involved in sperm function of normozoospermic and nonnormozoospermic individuals, paving the way for biomarkers of male infertility.

## Discussion

This study investigated the seminal plasma AAs of normozoospermic and non-normozoospermic groups to understand the potential role of AAs in male infertility. For the first time, using HPLC-LSF with high sensitivity and specificity, this approach enabled accurate detection and quantification of AAs even at very low concentrations, demonstrating sensitivity at the femtomole level [11]. Conventional methods for analyzing AAs, such as HPLC with UV or fluorescence detection, gas chromatography, and liquid chromatography coupled with mass spectrometry, all come with specific methodological challenges. These methods often have several limitations, including insufficient sensitivity for low-abundance amino acids, complex sample preparation, unstable derivatization, and high costs associated with instrumentation and maintenance [10]. Furthermore, seminal plasma is a complex biochemical fluid that contains proteins and salts, which frequently leads to signal suppression or peak interference in many traditional systems. In light of these challenges, HPLC with laser-stimulated fluorescence (HPLC-LSF) has been investigated as a sensitive analytical approach for detecting amino acids in seminal plasma. The HPLC-LSF system used in this study demonstrates femtomole detection limits, requires only trace amounts of sample, and enables improved selective separation of AAs without the need for expensive instrumentation.

The univariate analysis revealed a significant difference between the two groups for all 17 AAs, except for aspartate. In general, the relative abundance of AAs was lower among the non-normozoospermic group compared to the normozoospermic groups. Similar trends have been reported in previous studies where higher levels of AAs were observed in normozoospermic men. Deng et al. examined the AA profiles in the seminal plasma of asthenozoospermic and oligozoospermic ejaculates and found a noticeable decline in comparison to normozoospermic men [8]. Further, decreased levels of seminal plasma AAs were found in azoospermic and idiopathic oligoasthenoteratozoospermic men [22]. The lower levels of AAs could be attributed to altered metabolic activity, resulting in reduced synthesis or increased utilization of AAs. These findings demonstrate a noteworthy connection between AAs and male infertility.

Further incorporation of PCA and PLS-DA effectively differentiated normozoospermic and non-normozoospermic samples. This analysis revealed distinct clusters and identified key AAs responsible for this clustering. Notably, BCAAs, alanine, serine, and histidine ranked the highest in terms of their VIP scores, highlighting their significance in sperm function. Interestingly, correlation analysis revealed associations between individual AAs and key sperm characteristics, including sperm count, motility, morphology, and DNA integrity. Notably, most AAs displayed a moderate positive correlation with sperm concentration, suggesting that these AAs may be linked to the processes involved in spermatogenesis and sperm capacitation. Additionally, a weak negative correlation was noted with BCAAs, glycine, serine, alanine, and phenylalanine with SCD test results. This implies a potential connection with sperm DNA integrity. These associations can be considered biologically relevant based on previous studies. Intermediates produced during BCAA catabolism participate in the TCA cycle and may influence mitochondrial ATP production and efficiency, potentially contributing to regulated reactive oxygen species generation and helping to prevent excessive oxidative damage [23]. Glycine, a precursor of glutathione, has also been reported to play a role in antioxidant pathways. Experimental studies have demonstrated that glycine supplementation can lower oxidative stress markers, improve mitochondrial function, and enhance sperm motility in animal models [24]. Furthermore, glycine combined with vitamin E has been associated with improved post-thaw sperm quality, increased antioxidant activity, and enhanced mitochondrial activity in livestock semen preservation, suggesting a potential role in reducing oxidative damage [25]. Serine plays a vital role in one-carbon metabolism through serine hydroxymethyltransferase, which supplies one-carbon units necessary for the synthesis and repair of nucleotides. It also regenerates cysteine for glutathione (GSH) through transsulfuration [26]. These metabolic functions help explain the observed relationships between serine levels and sperm DNA integrity. Similarly, there is a moderate correlation between these AAs and sperm motility, supporting likely due to their involvement in energy-related metabolic processes and cellular redox homeostasis [4]. The BCAAs (leucine, isoleucine, and valine) are intermediates that enter the TCA cycle, ultimately promoting ATP production. The TCA cycle is directly fueled by the acetyl-CoA and succinyl-CoA produced during BCAA metabolism [27]. Since sperm motility relies on mitochondrial activity, changes in BCAA metabolism can influence motility characteristics [28]. Glutamate, one of the most common intracellular AAs, plays a crucial role in redox balance within cells by facilitating NAD +/NADH cycling [29]. This process is essential for maintaining oxidative homeostasis in sperm cells. Lysine, another essential AA, has been observed to undergo acetylation in various metabolic enzymes within sperm [30]. Since mature sperm are transcriptionally silent, post-translational modifications like lysine acetylation are believed to be important for regulating metabolic activity and energy expenditure, both of which are critical for motility and fertilization [31]. Alanine is a nonessential AA that undergoes transamination to pyruvate, which is then converted into acetyl-CoA and enters the TCA cycle. This links alanine metabolism to cellular energy pathways through the glucose-alanine cycle [32]. Furthermore, previous studies have reported that the metabolism of glycine, serine, and threonine is associated with sperm motility, the acrosome reaction, and mitochondrial defense mechanisms [33].

The ROC curve analysis demonstrated good discriminatory performance for the combination of five AA biomarkers: histidine, leucine, alanine, serine, and valine, which achieved an AUC of 0.96. This indicates that the identified AA panel may have potential utility in differentiating between the study groups within the analyzed cohort. Combining these metabolic markers with standard semen analysis may provide insights into the complexities of sperm dysfunction, particularly in cases of borderline or unexplained infertility. Future validation in larger independent cohorts is essential to confirm the reliability of this method and assess its clinical relevance. Furthermore, pathway analysis revealed nine statistically significant metabolic pathways with phenylalanine, tyrosine, and tryptophan biosynthesis showing the highest impact scores. These pathways have been associated with redox homeostasis and mitochondrial activity, both of which are important in sperm physiology. Phenylalanine serves as a precursor for catecholamines (neurotransmitters), including dopamine and norepinephrine, which are known to influence mitochondrial oxidative phosphorylation [34]. This process could indirectly influence ATP generation, which is essential for sperm motility, capacitation, and the acrosome reaction. Similarly, tryptophan has been shown to enhance sperm motility and lifespan in vitro [35]. These results indicate that certain AAs and metabolic pathways are associated with different parameters of sperm quality. These findings emphasize the metabolic aspects of male fertility and generate hypotheses for future functional studies aimed at further understanding the mechanistic roles of AAs in sperm physiology.

## Conclusion

Amino acid profiling is a promising method for investigating the metabolic differences associated with male infertility. Integrating metabolic profiling with the clinical assessment of semen parameters may allow andrologists and clinicians to move beyond conventional biomarkers and toward a more comprehensive interpretation of the underlying biological mechanisms contributing to sperm pathologies. However, the present study has some limitations that should be considered, particularly concerning sample size and group distributions. First, the total sample size, especially among specific non-normozoospermic subjects, was relatively small, resulting in low statistical power to identify significant differences, particularly concerning AAs with moderate effect sizes. This may have led to overlapping AA profiles across the different non-normozoospermic categories, underscoring the need for larger, more evenly distributed cohorts in future research.

Investigations on sperm motility characteristics of Makler counting chamber are not recommended in WHO 2021. However, the andrology laboratory has been using it routinely with adequate quality control measures. Additionally, data about the participants’ diets and nutritional intake were not collected. Since most essential AAs are obtained through diet, individual dietary differences could have influenced the AA composition of seminal plasma. While testicular and accessory gland metabolism also plays a role in shaping seminal plasma composition, the absence of dietary data represents a potential confounding variable. Further, the study subjects are the male partners of infertile couples who underwent semen analysis as a part of the primary investigation, and hence, we could not get detailed metadata. However, information on chronic illness, long-term medication details was collected, but no evident changes were observed across the groups. Future studies should aim for larger, more evenly distributed sample sizes, along with independent subgroup analyses, to enhance our understanding of the metabolic changes associated with different semen profiles. Despite these limitations, this study has made valuable preliminary observations on changes in the seminal plasma AA profile associated with non-normozoospermic semen characteristics, using the sensitive HPLC-LIF platform.

## Supplementary Information

Below is the link to the electronic supplementary material.

ESM 1 (PNG 377 KB)

High Resolution Image (TIF 246 KB)

ESM 2 (PNG 339 KB)

High Resolution Image (TIF 217 KB)

ESM 3 (PNG 334 KB)

High Resolution Image (TIF 217 KB)

ESM 4 (PNG 296 KB)

High Resolution Image (TIF 195 KB)

ESM 5 (PNG 319 KB)

High Resolution Image (TIF 218 KB)

ESM 6 (PNG 315 KB)

High Resolution Image (TIF 216 KB)

ESM 7 (PNG 271 KB)

High Resolution Image (TIF 188 KB)

ESM 8 (DOCX 18.0 KB)

ESM 9 (DOCX 19.6 KB)

## Data Availability

No datasets were generated or analysed during the current study.
