# Supplementary material for: Novel HPLC–laser-stimulated fluorescence profiling of seminal plasma amino acids linked to ejaculate quality
Source: J Assist Reprod Genet. 2026 Jul 2;43(8):2543–55. doi: 10.1007/s10815-026-03944-9 (PMC13598000; doi:10.1007/s10815-026-03944-9)
Supplement: Supplementary file 1 [file 10815_2026_3944_MOESM8_ESM.docx]

**Supplementary Table S1. Age and sperm characteristics of the study subjects**

|  | Normozoospermic  (n=30) | Non-normozoospermic  (n=51) | p-value |
| --- | --- | --- | --- |
| Age (years) | 37.1±4.5 | 36.1±4.5 | 0.524 |
| Volume (mL) | 3.1±1.4 | 2.9±1.8 | 0.621 |
| Sperm concentration (10^6^/mL) | 46.4±21.2 | 5.8±4.3 | < 0.001 |
| Total motility (%) | 66.0±11.9 | 46.7±19.3 | < 0.001 |
| Progressive motility (%) | 46.3±15.2 | 27.1±18.6 | < 0.001 |
| Normal morphology (%) | 13.7±5.2 | 8.4±5.5 | < 0.001 |
| Vitality (%) | 68.5±12.5 | 55.6±15.8 | < 0.001 |
| DNA fragmented spermatozoa (%) | 22.5±4.5 | 34.8±14.2 | < 0.001 |

*Values are expressed in Mean±SD*
