# Supplementary material for: Novel HPLC–laser-stimulated fluorescence profiling of seminal plasma amino acids linked to ejaculate quality
Source: J Assist Reprod Genet. 2026 Jul 2;43(8):2543–55. doi: 10.1007/s10815-026-03944-9 (PMC13598000; doi:10.1007/s10815-026-03944-9)
Supplement: Supplementary file 2 [file 10815_2026_3944_MOESM9_ESM.docx]

Table S2. Intensity variation of seminal plasma amino acids in specific ejaculate types.

| **Amino Acids** | **N^a^ (n= 30)** | | **O^b^ (n= 33)** | | **OA^c^ (n= 11)** | | **OAT^d^ (n= 07)** | | | **Significant groups (p<0.05)** |
| --- | --- | --- | --- | --- | --- | --- | --- | --- | --- | --- |
|  | **Median** | **IQR** | **Median** | **IQR** | **Median** | **IQR** | **Median** | | **IQR** |  |
| Aspartate | 0.01 | 0 | 0.01 | 0.01 | 0.000 | 0.010 | 0.01 | 0.01 | | a:c, a:d |
| Glutamate | 0.2 | 0.0725 | 0.12 | 0.08 | 0.080 | 0.085 | 0.09 | 0.03 | | a:b, a:c, a:d |
| Hydroxyproline | 0.32 | 0.0875 | 0.2 | 0.11 | 0.140 | 0.055 | 0.17 | 0.11 | | a:b, a:c, a:d |
| Glutamine | 0.245 | 0.075 | 0.15 | 0.12 | 0.110 | 0.070 | 0.15 | 0.1 | | a:b, a:c, a:d |
| Serine | 0.74 | 0.103 | 0.5 | 0.21 | 0.460 | 0.165 | 0.25 | 0.215 | | a:b, a:c, a:d |
| Threonine | 0.25 | 0.08 | 0.16 | 0.09 | 0.130 | 0.060 | 0.14 | 0.08 | | a:b, a:c, a:d |
| Glycine | 0.18 | 0.035 | 0.11 | 0.06 | 0.100 | 0.050 | 0.1 | 0.055 | | a:b, a:c, a:d |
| Alanine | 0.775 | 0.14 | 0.54 | 0.19 | 0.430 | 0.145 | 0.36 | 0.175 | | a:b, a:c, a:d |
| Proline | 0.295 | 0.095 | 0.18 | 0.07 | 0.220 | 0.105 | 0.14 | 0.06 | | a:b, a:c, a:d |
| Valine | 0.6 | 0.14 | 0.36 | 0.2 | 0.300 | 0.125 | 0.33 | 0.26 | | a:b, a:c, a:d |
| Tryptophan | 0.035 | 0.01 | 0.02 | 0.01 | 0.030 | 0.025 | 0.01 | 0.01 | | a:b; a:d |
| Isoleucine | 0.705 | 0.16 | 0.5 | 0.15 | 0.360 | 0.110 | 0.44 | 0.23 | | a:b, a:c, a:d |
| Leucine | 0.845 | 0.165 | 0.57 | 0.26 | 0.400 | 0.145 | 0.5 | 0.3 | | a:b, a:c, a:d |
| Phenylalanine | 0.33 | 0.0675 | 0.2 | 0.14 | 0.170 | 0.045 | 0.22 | 0.13 | | a:b, a:c, a:d |
| Cysteine+Cystine | 0.02 | 0.01 | 0.01 | 0 | 0.010 | 0.000 | 0.02 | 0.01 | | a:b |
| Lysine | 0.19 | 0.0475 | 0.09 | 0.08 | 0.080 | 0.050 | 0.08 | 0.04 | | a:b, a:c, a:d |
| Histidine | 0.945 | 0.185 | 0.54 | 0.25 | 0.470 | 0.175 | 0.6 | 0.33 | | a:b, a:c, a:d |

*(IQR-Interquartile range; N-Normozoospermic; O-Oligozoospermic; OA-Oligoasthenozoospermic; OAT-Oligoasthenoteratozoospermic samples).*
